# Erythrocyte compression index is impaired in patients with residual vein obstruction

**DOI:** 10.1007/s11239-018-1650-1

**Published:** 2018-03-27

**Authors:** Michal Zabczyk, Joanna Natorska, Anetta Undas

**Affiliations:** 1 Institute of Cardiology, Jagiellonian University Medical College, 80 Pradnicka St., 31-202 Kraków, Poland; 2 Krakow Centre for Medical Research and Technologies, John Paul II Hospital, Kraków, Poland

**Keywords:** Anticoagulation, Clot contraction, Fibrin clot, Clot lysis time, Residual vein obstruction

## Abstract

Defective clot contraction has been postulated to contribute to thrombosis. We aimed to evaluate the association of residual vein obstruction (RVO) with erythrocyte compression within the whole-blood clot. We studied 32 patients with venous thromboembolism (VTE) taking vitamin K antagonists (VKAs) for at least 3 months (median time in therapeutic range 60%), including 12 (37.5%) with RVO, and 32 age- and sex-matched controls. In all study participants we evaluated whole blood clot retraction, expressed as the erythrocyte compression index (ECI), defined as a ratio of mean polyhedrocyte area to mean native erythrocyte area, along with clot area covered by polyhedrocytes, plasma clot permeability (Ks), clot lysis time (CLT), and thrombin generation. In both groups higher ECI, indicating impaired clot contraction, increased with older age, higher body mass index, red blood cell distribution width, and lower platelet count (all p < 0.05), but not with red blood cell count. In VTE patients ECI was 15.8% higher than in controls (median 63.6 vs. 54.9%, p = 0.021). Subjects with RVO had 20% higher ECI and 155% lower clot area covered by polyhedrocytes. RVO patients had also prolonged CLT by 41%, but not Ks, and elevated peak thrombin generation by 33%, as compared to those without RVO (all p < 0.05). This study is the first to show impaired compression of erythrocytes in RVO patients despite VKA anticoagulation. Altered ECI coexisted with hypolysability and increased thrombin generation. ECI might be useful in the diagnostic process of RVO or post-thrombotic syndrome and can help optimize the anticoagulant therapy.

**Electronic supplementary material:**

The online version of this article (10.1007/s11239-018-1650-1) contains supplementary material, which is available to authorized users.

## Highlights

- The erythrocyte compression index (ECI) is an novel measure of clot contraction
- Higher ECI coexists with increased thrombin generation and hypofibrinolysis
- ECI increases in patients with residual vein obstruction despite anticoagulation
- A prognostic value of clot contraction should be established

## Introduction

During clot formation, thrombin burst activates platelets and converts fibrinogen into fibrin. Fibrin sticks to the activated platelets via the integrin receptor αIIbβ3 to form a platelet–fibrin meshwork comprising the structural basis of a hemostatic clot or an obstructive thrombus [1]. When the blood clot is formed in vitro in a tube, it undergoes volume shrinkage, and liquid serum is around compressed clot [2]. This process is called clot contraction or retraction [3]. Cellular blood components, in particular red blood cells (RBCs), platelets, and plasma fibrinogen concentration influence the rate and extent of clot contraction [3]. Reduced clot contraction was associated with a lower platelet count and/or their dysfunction, elevated hematocrit, leukocytosis, increased plasma fibrinogen, and other changes in blood composition that may affect platelet function and properties of blood clots [4]. It has been postulated that defective clot retraction might contribute to a tendency to thrombosis [5]. Contraction of blood clots has been suggested to be involved in the restoration of blood flow in arteries [6]. Tutwiler et al. [4] have demonstrated impaired clot contraction in patients with acute ischemic stroke associated with platelet dysfunction [7].

Compressed erythrocytes, called polyhedrocytes due to their unique polyhedral shape, have been found in human whole blood clots generated in vitro and in human intracoronary thrombi [3, 8]. Polyhedrocyte formation contributes to the restoration of blood flow in occluded vessels [3, 5]. Spontaneous restoration of blood flow occurs in venous thromboembolism (VTE), but the extent and rate of this process have not been reported yet. Residual vein obstruction (RVO) is observed despite the anticoagulant therapy in 50% of deep vein thrombosis (DVT) patients and its risk is increased in the case of suboptimal anticoagulant therapy, delayed diagnosis, iliac vein involvement, unprovoked DVT, and thrombophilia [9–11]. Prolonged clot lysis time (CLT) and reduced fibrin clot permeability (K_s_) have been found to be risk factors for RVO [12]. It is unknown whether RVO is associated with impaired clot contraction. We hypothesized that patients with RVO display impaired compression of erythrocytes. To address this issue, we developed a new approach to assess clot contraction based on the microscopic RBC compression in VTE patients using the erythrocyte compression index (ECI).

## Materials and methods

We enrolled 32 patients with DVT treated with vitamin K antagonists (VKAs) for at least 3 months and 32 apparently healthy controls with no history of VTE matched for age and sex. The diagnosis of DVT was established based on a positive finding of color duplex sonography. The exclusion criteria were as follows: arterial or venous thromboembolic events within previous 6 months, known cancer, signs of acute infection, chronic inflammatory disorders, liver injury, estimated glomerular filtration rate (eGFR) < 30 mL/min, pregnancy. Venous thromboembolism patients were eligible if they declared regular VKA intake and provided INR values measured at least monthly [13]. Time in therapeutic range (TTR) was assessed by the Rosendaal method [14]. At enrollment we assessed whether or not there was RVO, defined as a vein transverse diameter greater than 2 mm or as residual thrombus occupying more than 40% of the vein area at maximum compressibility [9]. The Jagiellonian University Ethical Committee approved the study and all the participants provided their written informed consent.

### Routine laboratory investigations

Fasting venous blood was drawn between 7 a.m and 10 a.m. and was kept at a room temperature. Blood samples were collected into citrated tubes (9:1 of 0.106 M sodium citrate), centrifuged at 2500×*g* and 20 °C for 10 min, snap-frozen within 60 min, and stored in small aliquots at − 80 °C until analysis. Complete blood count including white blood cells (WBC), RBC, hemoglobin, hematocrit, RBC distribution width (RDW), platelet count and platelet distribution width (PDW) were assayed using the hematological analyzer Sysmex XT2000i (Sysmex Corporation, Kobe, Japan). Fibrinogen was assessed using the Clauss method. All patients with VTE were screened for thrombophilia. Plasma levels of factor (F)VIII were evaluated using FVIII-deficient substrate plasma and the Behring coagulation system (Siemens Healthcare Diagnostics, Marburg, Germany).

### Preparation of whole blood clots

The clot size after retraction was measured as previously described with slight modifications [2]. Clotting was initiated by addition of 40 µL of activation mixture (CaCl_2_ and human thrombin at final concentrations of 0.01 M and 1 U/mL, respectively) to 960 µL of prewarmed for 5 min at 37 °C citrated whole blood. Samples were incubated at 37 °C for 1 h and the clot size was measured as a difference between the initial sample volume and the fluid volume around the clot after its retraction and was expressed as a percentage. To provide new insights into the in vitro clot contraction we performed analysis of erythrocyte compression inside the whole blood clot. Clotting was initiated using the similar model as for the clot size measurement in a final volume of 50 μL.

### Scanning electron microscopy

**Fig. 1:**
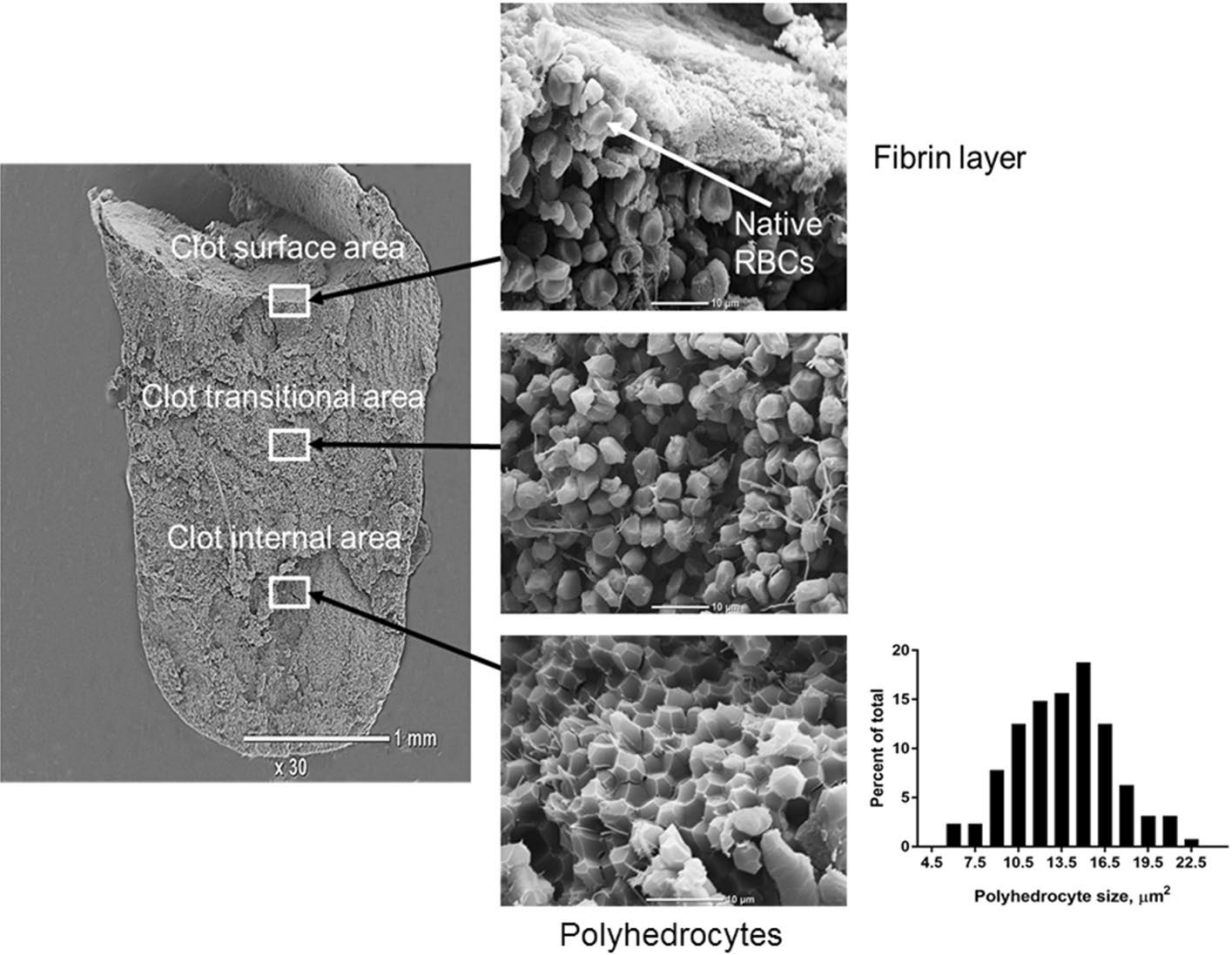
A representative SEM image of a retracted whole blood clot (magnifications ×30 and ×3600) used for semiquantitative analysis of polyhedrocytes content and size measurement in 40 selected areas located in three vertical axes (from the left to the right and from the top to the bottom of the clot)

**Fig. 2:**
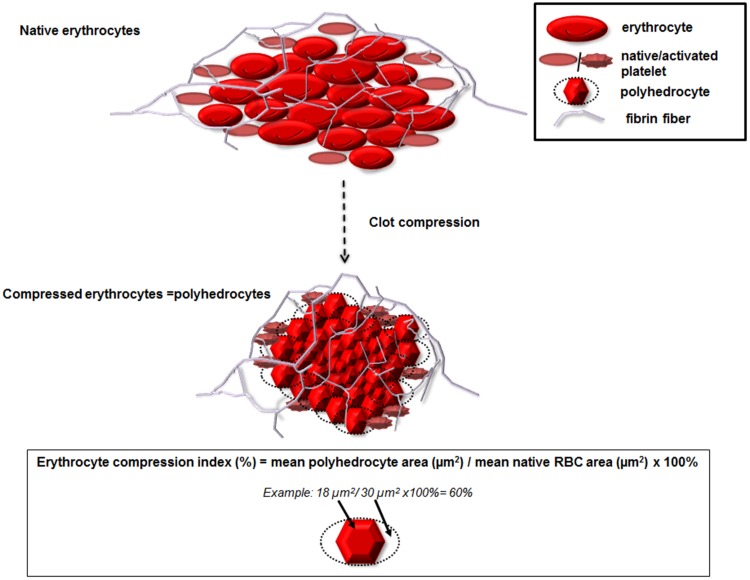
A scheme of erythrocytes compression to its polyhedral form during in vitro clot formation

Scanning electron microscopy (SEM) was performed as previously described [15]. Blood clots were washed in 0.1 M NaCl and fixed in 2.5% glutaraldehyde, dehydrated by a graded series of ethanol concentrations and frozen in *tert*-butyl alcohol for 2 h. Then, clots were dried in a vacuum and coated with gold. Microphotographs were acquired using a scanning electron microscope (JEOL JCM-6000, Japan). We analyzed 40 images (Fig. 1) for each clot and then assessed, using ImageJ (US National Institutes of Health), the area (µm^2^) of native RBCs (mostly observed within the clot surface area) and polyhedrocytes (mostly located in the internal parts of the clot). Results were presented as means ± standard deviations (SD) of about 100 consecutive RBC areas obtained from SEM images. ECI was defined as a ratio of the mean polyhedrocyte area to the mean native RBC area expressed as a percentage (Fig. 2). The inter- and intra-observer agreement was 93 and 95%, respectively.

### Fibrin clot characteristics and thrombin generation

Plasma fibrin clot parameters were measured as described previously [16]. Briefly, K_s_ was assessed using a pressure-driven system, which indicates the pore size in fibrin networks. CLT induced by tissue plasminogen activator added to plasma with thrombin was determined. Calibrated automated thrombography was used to measure thrombin generation, as described [17]. For details see the Supplementary Material.

### Statistical analysis

The study was powered to have a 90% chance of detecting a 10% difference in clot contraction using a p value of 0.01, based on the previous study [4]. In order to demonstrate such a difference or greater, at least five patients were required in each group. Categorical variables are presented as numbers and percentages. Continuous variables are expressed as mean ± SD or median and interquartile range (IQR). Normality was assessed by the Shapiro–Wilk test. Differences between groups were compared using the Student’s for normally distributed variables and the Mann–Whitney U test was used for non-normally distributed continuous variables. Categorical variables were compared by the Fisher’s exact test. The Spearman’s rank correlation coefficient was computed to measure the association between two variables. A two-sided p < 0.05 was considered statistically significant. All calculations were done with STATISTICA Version 12.5 (StatSoft Inc., Tulsa, OK, USA).

## Results

### Patient characteristics

The VTE patients and control subjects were well matched (Table 1). Higher body mass index (BMI) was, however, observed in the former group. Factor V Leiden mutation was diagnosed in 6 (18.8%) VTE patients, while elevated FVIII > 150% was observed in 13 (40.6%) patients. Among the VTE patients, 20 (62.5%) subjects were treated with warfarin and 12 (37.5%) with acenocoumarol with a median TTR of 60% (IQR 55–80%). In 18 patients (56%) INR was between 2 and 3 [median 2.44 (2.18–2.77)], while in 2 patients (6%) INR was supratherapeutic and in 12 (37%) INR was below two on the day of blood draw. VTE patients had higher fibrinogen, CRP, and triglycerides, but not platelet count or RBCs (Table 1). VTE patients taking VKAs had 23.7% lower K_s_, 12.4% longer CLT, 17.7% lower ETP, and 44.8% higher peak thrombin generation compared to controls (Table 1). The differences remained significant after adjustment for fibrinogen.

**Table 1:** Characteristics of the study participants

Variables	VTE patients (n = 32)	Controls (n = 32)	p value	VTE patients with RVO (n = 12)	VTE patients without RVO (n = 20)	p value
Age (years)	38.5 (33.5–47.5)	39.0 (29.5–43.0)	0.41	36.0 (33.0–48.5)	39.5 (35.0–45.5)	0.74
Male [n (%)]	16 (50)	17 (53)	0.80	7 (58.3)	9 (45)	0.72
BMI (kg/m^2^)	28.0 ± 6.4	24.4 ± 4.9	0.036	27.6 (24.6–30.0)	28.4 (23.4–31.1)	0.98
Current smoking [n (%)]	5 (15.6)	3 (9.4)	0.45	4 (33.3)	1 (5)	0.053
Laboratory measurements						
INR	2.16 ± 0.86	1.02 ± 0.07	< 0.0001	2.02 (1.44–2.30)	2.34 (1.47–2.77)	0.22
aPTT (s)	26.2 (24.5–27.3)	35.7 (29.3–39.6)	< 0.0001	31.6 (27.1–38.4)	36.2 (31.7–41.0)	0.14
RBC (10^6^/µL)	4.84 ± 0.44	4.77 ± 0.34	0.48	4.92 (4.70–5.40)	4.63 (4.41–5.02)	0.11
WBC (10^3^/µL)	6.47 (5.3–8.3)	5.95 (5.4–6.6)	0.17	6.5 (5.7–8.8)	7.1 (4.9–8.3)	0.60
Hemoglobin (g/dL)	14.2 ± 1.5	14.2 ± 1.0	0.95	14.9 (13.7–15.6)	13.6 (12.8–14.6)	0.044
Hematocrit (%)	42.4 ± 3.6	42 ± 2.6	0.58	44.2 (40.1–46.3)	40.3 (39.2–42.9)	0.040
RDW (%)	13.3 (12.9–14.2)	12.5 (12.2–13.0)	< 0.0001	13.1 (12.8–13.5)	13.5 (13.0–14.5)	0.17
Platelets (10^3^/µL)	269 (219–292)	241 (212–279)	0.22	251 (217–284)	271 (222–294)	0.52
PDW (%)	12.8 ± 2.1	12.9 ± 1.7	0.82	13.3 (11.0–14.5)	12.2 (11.1–13.6)	0.45
Creatinine (µM)	74 ± 10	79 ± 12	0.13	72 (68–78)	74 (64–81)	0.95
Glucose (mM)	5.4 ± 0.9	5.0 ± 0.5	0.055	5.2 (4.5–5.5)	5.3 (5.1–5.8)	0.40
Fibrinogen (g/L)	3.05 (2.86–3.64)	2.53 (2.27–3.20)	0.017	3.04 (2.89–3.58)	3.06 (2.77–3.93)	0.83
CRP (mg/L)	3.6 (1.0–6.4)	1.5 (0.6–1.4)	0.0022	2.2 (0.6–5.0)	4.2 (2.0–6.8)	0.20
Total cholesterol (mM)	5.16 (4.36–5.72)	4.56 (4.14–5.19)	0.22	5.16 (4.30–5.90)	5.19 (4.43–5.64)	0.99
LDL-C (mM)	2.92 (2.52–3.67)	2.86 (2.35–3.52)	0.43	2.92 (2.36–3.68)	3.23 (2.54–3.61)	0.94
HDL-C (mM)	1.62 ± 0.45	1.62 ± 0.37	0.74	1.57 (1.15–2.03)	1.61 (1.24–1.76)	0.77
TG (mM)	1.27 (0.95–1.78)	0.91 (0.66–1.14)	0.0026	1.58 (0.94–2.75)	1.27 (0.95–1.73)	0.53
K_s_ × 10^− 9^cm^2^	5.52 ± 1.26	7.23 ± 1.09	< 0.0001	5.12 (4.25–5.59)	5.43 (4.6–6.72)	0.063
CLT (min)	109 ± 22	97 ± 14	0.012	135 (103–168)	96 (77–138)	< 0.0001
Lag time (min)	3.3 ± 1.4	5.4 ± 1.6	< 0.0001	3.2 (2.4–4.3)	3.3 (4.4–4.5)	0.78
ETP (nM × min)	1258 ± 408	1474 ± 146	< 0.01	1241 (910–1409)	945 (521–1382)	< 0.01
Peak thrombin generation ( nM)	294 ± 169	203 ± 64	< 0.0001	302 (161–361)	227 (98–317)	0.032
Time to peak thrombin (min)	5.0 ± 1.5	8.2 ± 2.5	< 0.0001	5.1 (4.0–7.1)	5.3 (4.1–7.3)	0.81

Values are given as mean ± standard deviation or a median (interquartile range), or number (percentage)

*aPTT* activated partial thromboplastin time, *BMI* body mass index, *CRP* C-reactive protein, *INR* international normalized ratio, *LDL-C* low density lipoprotein cholesterol, *HDL-C* high-density lipoprotein cholesterol, *RVO* residual vein obstruction, *TG* triglycerides, *WBC* white blood cells, *K*_s_ fibrin clot permeability, *CLT* clot lysis time, *ETP* endogenous thrombin generation

### Erythrocyte compression index

The new parameter describing compression of erythrocytes, ECI, within the whole blood clot was strongly associated with the clot size assessed using the previously established method (r = 0.44, p < 0.01 for the control group and r = 0.58, p < 0.001 for anticoagulated patients). The clot size did not differ between control subjects and VTE patients [54 (50–59.5) vs. 55 (50–63.5)%, p = 0.66, respectively]. However, ECI was 15.8% higher in VTE patients taking VKAs than in controls indicating impaired compression of erythrocytes [63.8 (58.7–69.0) vs. 54.0 (50.0–59.5)%, p = 0.021, respectively].

In controls and VTE patients, ECI was related with age, BMI, platelet count (Supplementary Fig. 1a–c), and RDW (r = 0.47, p = 0.023 and r = 0.53, p = 0.011, respectively), but not with RBC count (p > 0.05).

The highest ECI was observed in anticoagulated patients with plasma fibrinogen between 3.0 and 4.0 g/L (median 66.5%) compared with those with fibrinogen between 2.0 and 3.0 g/L (median, 61.8%) and those below 2.0 g/L (median 55.7%) (p < 0.05 for ANOVA). ECI was inversely associated with a platelet (× 10^3^/µL)/fibrinogen (g/L) ratio in VTE patients (r = − 0.58, p = 0.0013). There were no differences in the ECI values regarding sex, smoking, FVIII, and CRP (all p > 0.05) in both groups. The clot area covered by polyhedrocytes [21.3 (8.6–45.0)% in the VKA group vs. 30 (15–50) % in controls; p = 0.24] was similar in both groups. The median polyhedrocyte size was 8.2% larger in VTE patients than in controls [17.1 (15.3–18.5) vs. 15.8 (10.6–17.0) µm, p = 0.037, respectively].

### RVO

**Fig. 3:**
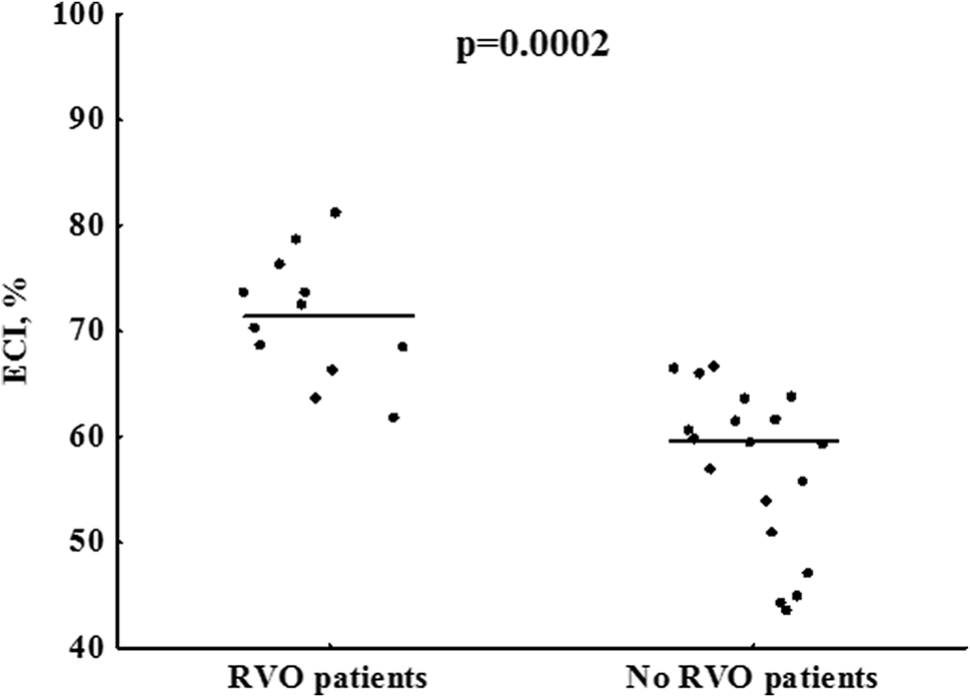
ECI in patients with or without residual vein obstruction (RVO). Horizontal lines denote medians

Residual vein obstruction was diagnosed in 12 (37.5%) DVT patients. RVO patients did not differ from the remainder with regard to demographic or routine laboratory variables, including fibrinogen and platelet count, except slightly higher hemoglobin and hematocrit in the former group (Table 1). Subjects with RVO had 20% higher ECI (Fig. 3), also after adjustment for hemoglobin or hematocrit, and 155% lower clot area covered by polyhedrocytes [12.5 (5.0–17.5) vs. 31.9 (15.0–52.5)%, p = 0.025]. The clot size however did not differ between patients with and without RVO (p > 0.05). RVO patients had also prolonged CLT by 41%, but not K_s_, and elevated ETP by 31.3% and peak thrombin generation by 33%, as compared to those without RVO (Table 1).

## Discussion

This study is the first to show that the clot contraction is impaired in patients with RVO despite anticoagulation. We provided evidence for the concept that the clot contraction contributes to the restoration of blood flow in veins and its impairment predisposes to RVO. Our findings suggest that contraction of clots might have identify patients at risk of such common complication of DVT and possibly also at risk of recurrent ipsilateral thrombosis [18]. Formation of deep venous thrombi is a dynamic process, which in 45% of subjects results in incomplete vein recanalization within 6–9 months of thrombosis [19]. Most of these changes occur during the first 3 months after DVT, while the clot structure and function at the DVT onset can predict recurrent thrombosis and its outcome [20]. We hypothesize that the clot ability to contract is a characteristic blood feature of each patient and can undergo modulation mediated by transient factors [21], however even after a few months since thrombosis and on anticoagulation, impaired clot contraction can be observed and influences the fate of thrombotic material in the vascular lumen.

From a methodological point of view, we have introduced the novel parameter describing the extent of RBCs compression within the clot, i.e. the ECI. Although this variable appears to have no clinical utility, at least for now, it may facilitate assessment of some modulators of clot contraction, particularly in patients with thromboembolic disorders. In our study, ECI showed positive associations—in both VTE patients and controls—with older age, higher BMI, and RDW, suggesting that in these conditions clot contraction is impaired. We found also that ECI was inversely associated with platelet count, in both studied groups, while a correlation of ECI with fibrinogen was observed in VTE patients only. Tutwiler et al. [4] presented similar effects of different platelet counts and fibrinogen levels on clot contraction. Increased platelet count results in faster clot retraction depending on contractile forces generated by the platelet cytoskeleton [22], while fibrin is essential for transmitting those forces between platelets [3]. Our data suggest that the contraction of the whole blood clot depends also on the size of RBCs trapped within the fibrin network. Thus anisocytosis could be one of the factors facilitating clot contraction through better RBCs fitting. Higher RDW values were associated with adverse clinical outcomes in patients with heart failure, coronary artery disease, or stroke [23–25]. Very recent data showed that the platelet packing density can regulate platelet activation and thus determine the clot architecture and retraction [26]. Platelet volume, PDW, and RDW-platelet ratio have been shown to be associated with the no-reflow phenomenon and cardiovascular complications in patients with ST-elevation myocardial infarction [27, 28]. Moreover, inflammation might play an important role in increased RDW values by promoting the release of immature RBCs into the circulation [29], thus might contribute to the impaired clot contraction. ECI might be useful in some specific circumstances for example in subjects with iron deficiency [30] or thrombocythemia [31]. We hypothesize that erythrocytes of similar sizes are more likely to form polyhedrocytes, while different sizes of RBCs hamper this process. Therefore, increased RDW would result in poorer ability of erythrocytes to provide an impermeable seal, due to minimal interstitial space, to increase fibrinolysis resistance.

During the clot formation, increasing levels of thrombin and/or fibrinogen have been shown to alter fibrin clot properties [32, 33]. We found that ECI was associated rather with the platelet/fibrinogen ratio, than with platelet count or fibrinogen alone, which is in line with the study by Cines et al. [3], who showed that at low fibrinogen levels a higher platelet count is required. This observation highlights a platelet-driven, fibrin-mediated mechanism of clot contraction [34]. The association of clot contraction with fibrin plasma clot lysability deserves a comment. Carroll et al. [5] showed that platelet-mediated clot contraction facilitated clot lysis suggesting an important effect of platelets in clot retraction. Our results extended this observation by showing that impaired clot contraction coexisted with prolonged CLT, which could be clinically relevant, since CLT has been reported as a risk factor for both venous and arterial thrombosis and recurrent VTE [16, 35–37]. It might be speculated that assessment of clot contraction, using ECI or possibly other measures can help identify individuals of risk of RVO. Given evidence for association between RVO and post-thrombotic syndrome as well as DVT recurrence, ECI might possibly be useful in the assessment and optimization of anticoagulant therapy. Further long-term follow-up studies are warranted to validate this hypothesis.

This study has several limitations. First, the sample size was limited and presented observations must be interpreted with caution. However, analyses were sufficiently powered and it is unlikely that the differences reported here result from a significant bias. Second, the relevance of clot contraction should be confirmed in vivo. However, impaired ECI in VTE patients with RVO supports our hypotheses. A prognostic value of clot contraction and its markers like ECI remains to be established.

The main finding of our work is that the whole blood clot contraction assessed in VTE patients with RVO is impaired even when assessed during anticoagulant therapy. Additionally, we found that altered clot contraction coexisted with increased thrombin generation and impaired susceptibility to fibrinolysis. Those data indicate that besides prothrombotic fibrin clot phenotype, clot contraction might be of major importance in thromboembolic disorders and regulation of this process appears to be much more complicated than one would have expected. Given a role of platelets in clot contraction and data shown by Rusak et al. [22] that tirofiban—a platelet glycoprotein IIb/IIIa inhibitor significantly reduced clot retraction, it seems that antiplatelet agents might improve clot retraction at least to some extent. Further studies are needed to clarify these complex associations.

## Electronic supplementary material

Below is the link to the electronic supplementary material.

Supplementary material 1 (DOCX 128 KB)
