# Supplementary material for: Erythrocyte compression index is impaired in patients with residual vein obstruction
Source: J Thromb Thrombolysis. 2018 Mar 27;46(1):31–8. doi: 10.1007/s11239-018-1650-1 (PMC5994218; doi:10.1007/s11239-018-1650-1)
Supplement: Supplementary file 1 — Supplementary material 1 (DOCX 128 KB) [file 11239_2018_1650_MOESM1_ESM.docx]

**Supplementary Material – Journal of Thrombosis and Thrombolysis**

**Erythrocyte compression index is impaired in patients with residual vein obstruction**

Michal Zabczyk^1^, Joanna Natorska^1,2^, and Anetta Undas^1,2^

1. Institute of Cardiology, Jagiellonian University Medical College, Krakow, Poland;
2. Krakow Centre for Medical Research and Technologies, John Paul II Hospital, Krakow, Poland

**Corresponding author:**

Anetta Undas, MD, PhD

Institute of Cardiology Jagiellonian University Medical College

80 Pradnicka St.,

31-202 Krakow, Poland

Tel. +48 12 6143004

Fax. +48 12 6142120

e-mail: [mmundas@cyf-kr.edu.pl](mailto:mmundas@cyf-kr.edu.pl)

**Materials and Methods**

Fibrin clot permeation was determined using a pressure-driven system [16]. Briefly, 20 mM calcium chloride and 1 U/mL human thrombin (Merck KGaA, Darmstadt, Germany) were added to 120 µL citrated plasma. After 2 hours of incubation in a wet chamber, tubes containing the clots were connected via plastic tubing to a reservoir of a buffer (0.01 M Tris, 0.1 M NaCl, pH 7.4) and its volume flowing through the gels was measured within 60 minutes. K_s_, which indicates the pore size, was calculated from the equation: K_s_ =Q×L×η/t×A×Δp, where Q is the flow rate in time t; L, the length of a fibrin gel; η, the viscosity of liquid (in poise); A, the cross-sectional area (in cm^2^), Δp, a differential pressure (in dyne/cm^2^) and t is percolating time. The interassay and intraassay coefﬁcients of variation were less than 8%.

To assess efficiency of clot lysis, we used the method described previously [16]. Briefly, 75 µL of assay buffer (5 mM TRIS-HCl, 15 mM NaCl with 1 mg/mL bovine serum albumin, pH 7.4) with addition of recombinant tPA (rtPA, Boehringer Ingelheim, Ingelheim, Germany) at a final concentration of 83 ng/mL and phospholipids (Rossix, Mölndal, Sweden) at a final concentration of 10 µM were added to 25 µL of citrated plasma in a microtiter plate. Then, 50 µL of a mixture of thrombin (final concentration 0.03 U/mL) and CaCl_2_ (final concentration 7.5 mM) were added with a multichannel pipette. The turbidity was measured at 405 nm at 37°C. CLT was defined as the time from the midpoint of the clear-to-maximum-turbid transition, which represents clot formation, to the midpoint of the maximum-turbid-to-clear transition (representing the lysis of the clot). The interassay and intraassay coefficients of variation were less than 8%.

Thrombin generation was assessed using the calibrated automated thrombogram (CAT; Thrombinoscope BV, Maastricht, the Netherlands) as previously described [17]. Briefly, 80 µl of thawed platelet poor plasma was mixed with 20 µl of a reagent containing recombinant relipidated TF and phospholipids, with the final concentrations of 5 pmol/l and 4 mmol/l, respectively. The reactions were performed in microtiter wells (Thermo Electron, Denmark) after automatic addition of a fresh starting reagent containing calcium chloride (100 mmol/l) and a thrombin specific fluorogenic substrate (Z-Gly-Gly-Arg-AMC) (2.5 mmol/l) in HEPES buffer. The fluorescence intensity was recorded by the Fluoroskan Ascent® microplate fluorometer (Thermo Fisher Scientific Oy, Vantaa, Finland) using the software program (Thrombinoscope BV, version 3.0.0.29). Each plasma sample was analyzed in duplicate, and the intraassay variability was 6%. The maximum concentration of thrombin formed during the recording time is described as the peak thrombin generation and the area under the curve represents endogenous thrombin potential (ETP). Lag-time is the time from the start of analysis until thrombin starts to generate.

**Figure I.** Associations of erythrocyte compression index (ECI) with age (A), body mass index (BMI; B), and platelet count (PLT; C) in anticoagulated patients (OAT; oral anticoagulat therapy) and controls.

**
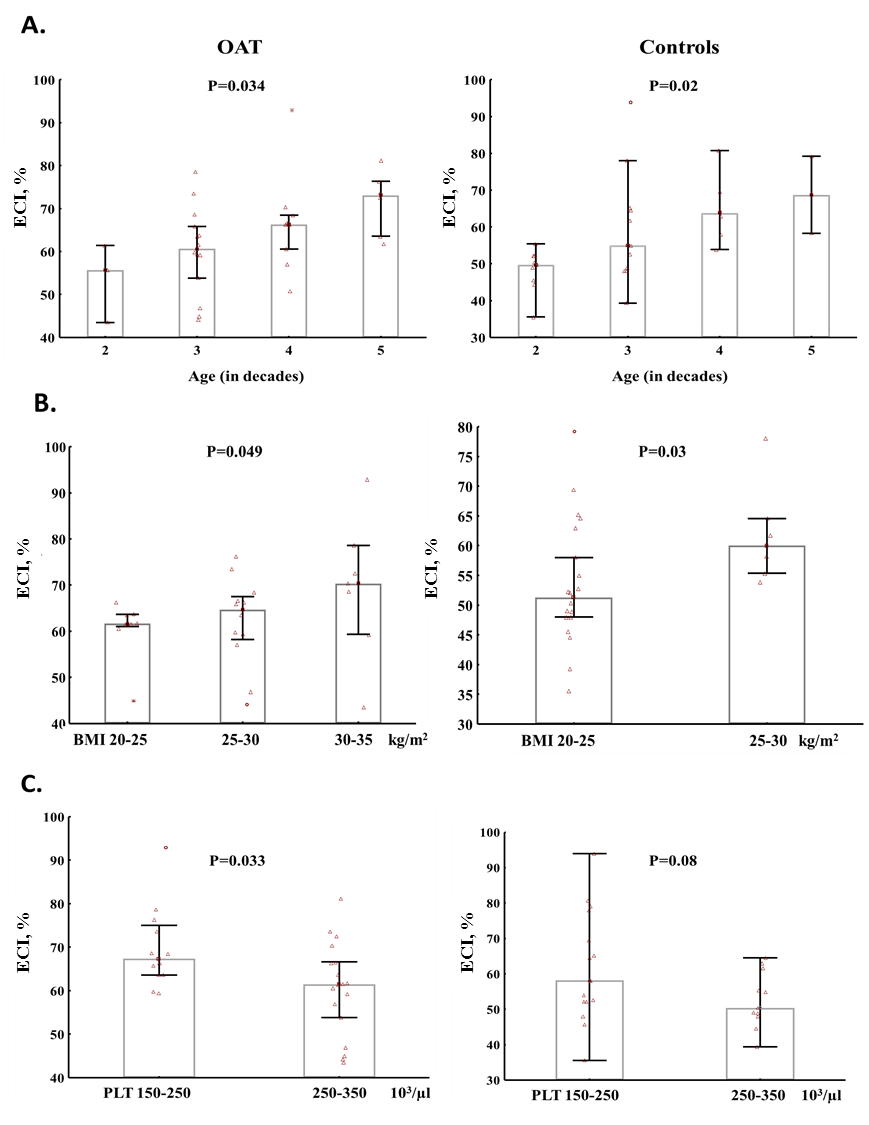
**
